# Assessing whether medical language is a barrier to receiving healthcare services in Bangladesh: an exploratory study

**DOI:** 10.3399/bjgpopen18X101641

**Published:** 2019-04-03

**Authors:** Badrul Alam Bhuiyan, Ishrat Jahan Urmi, Mahbub Elahi Chowdhury, Tajrian Rahman, Abu Syed Hasan, Padam Simkhada

**Affiliations:** 1 Health Researcher, Global Consortium for Public Health Research, Liverpool John Moores University, Liverpool, UK; 2 Junior Consultant, Ministry of Health, Government of Bangladesh, Dhaka, Bangladesh; 3 Health Researcher, Independent, Liverpool, United Kingdom; 4 Scientist and Director, Universal Health Coverage, icddr,b (International Centre for Diarrhoeal Disease Research), Dhaka, Bangladesh; 5 Founder Member, Global Consortium for Public Health Research, Liverpool John Moores University, Liverpool, UK; 6 Academic Researcher, Department of Pharmacy, Asia Pacific University, Dhaka, Bangladesh; 7 Senior Validation Executive, Validation Department, Eskayef Pharmaceuticals, Dhaka, Bangladesh; 8 Technical Officer, Family Planning & ASRH, UNFPA (United Nations Population Fund), Dhaka, Bangladesh; 9 Professor, International Public Health and Associate Dean for Global Engagement, Public Health Institute, Liverpool John Moores University, Liverpool, UK; 10 Co-ordinator, Global Consortium for Public Health Research, Liverpool John Moores University, Liverpool, UK

**Keywords:** General practice, medicine, medical-language, prescription, Bangladesh, Health services

## Abstract

**Background:**

In many global settings, medical language acts as a barrier to accessing and using health services. However, this issue remained unexplored in Bangladesh, where the non-native English language is commonly used for health care.

**Aim:**

To examine whether medical language is an obstacle for obtaining health services in Bangladesh and to provide policy recommendations.

**Design & setting:**

An exploratory study was undertaken to identify the impact of medical language on general practice. Data were collected online from Bangladeshi people between July–November 2014.

**Method:**

A semi-structured questionnaire was developed through Google Forms for data collection. The snowball technique was applied to obtain data purposively from 50 participants. With prior consent, the questionnaire along with the online link was sent to responders by email. When responders clicked on the 'submit' option of the questionnaire, responses were stored online automatically in the pre-built system. Quantitative data were analysed using SPSS (version 22). Textual data analyses (especially of suggestions of the responders) were conducted using a thematic approach.

**Results:**

Among study participants, 44% (*n* = 22) said that English language was the choice for writing prescriptions by health service providers in Bangladesh, and 26% said that a mixture of Bengali and English was used. Around 30% of the study participants could not understand medical language used by doctors (this includes those who were not sure or preferred not to say). Among responders, 78% said that medical language was affecting the treatment process and 48% were of the opinion that it was acting as a barrier in receiving health services.

**Conclusion:**

Medical language is acting as a barrier in the health services of Bangladesh. Tailored interventions must be developed and implemented to overcome medical language barriers in health services in order to strengthen the health system.

## How this fits in

No previous research was found that explored the impact of medical language on accessing and using health services in Bangladesh. This study explored for the first time how medical language acts as a barrier to obtaining effective health services in Bangladesh. In addition, some policy recommendations have been provided in this article to overcome the medical language barrier. This article will help clinicians to strengthen the doctor–patient relationship through effective communication, which can be scaled-up around the world.

## Introduction

Medical language is an important factor in health services all over the world. The language used in prescribing medicine has its own conventions of lexicon, discourse, phraseology, and abbreviations, which are different from the usual language of lay people.^1–3^ It is believed that English language (among 6000 global languages) is widely used for practising medicine globally, although many health service providers use their local language in prescriptions, along with other global languages.^1,4^ It is also impossible to deny the huge influence of ancient Greek medicine and the Latin language on medical terminology.^1,5^ This presents difficulties for effective health service provision in Bangladesh, where about 98% of people speak Bengali (also known as Bangla) and where English is the second official language.^6–9^ To tackle the preventable medical language barrier and, ultimately, to obtain a successful outcome from health services in Bangladesh, patients need to understand the language used by doctors or by other health service providers.

The medical language used in practising medicine and providing health services carries a special attribute because it is different from the native language people speak in their day-to-day conversations.^10,11^ As such, the language difference between doctors and patients sometimes becomes a strong barrier to achieving a successful treatment outcome.^12^ There is also strong evidence from around the world that has proven that miscommunication among health providers and patients plays a major role in the healthcare system, and that medical language is acting as a barrier to achieving an effective health service.^13–16^ Although no medical language-related research set in Bangladesh was found, a number of studies were conducted in other parts of the world that identified medical language as a barrier in healthcare provisions. Therefore, it is important to know the unexplored medical language issues related to health services in Bangladesh. It is also important to know if medical language acts as a barrier in Bangladesh, as there is a chance of miscommunication between lay patients and health service providers, and it is very difficult for a nation to get a successful outcome from the health services without overcoming the medical language barrier.^11,17^ It is evident through many studies conducted globally that medical language barriers not only lead to miscommunication with healthcare providers, but also can have deleterious effects on patients’ health.^18–21^

There is a dearth of data related to medical language and healthcare services in Bangladesh. An extensive literature review was performed to search for any previous relevant research, but unfortunately, until recently, no academic study could be found that explored the burning issue of the medical language barrier in Bangladesh. This article reports, for the first time, responders’ views regarding how medical language obstructs patients in Bangladesh in receiving health services, and what can be done to overcome this preventable problem. The study aimed to explore the languages that are being used for the general practice of medicine in Bangladesh, and to find out the impact of medical language on the treatment process. The study also aimed to explore how to overcome the medical language barrier hiding inside the healthcare system in Bangladesh.

## Method

An exploratory study was conducted using a semi-structured questionnaire that was developed through Google Forms. With prior consent, email addresses of the study participants were collected, and data were collected online using the snowball technique. The study questionnaire was sent to the responders by email (along with the website link for the online data collection). The responders were asked to share the email (containing the study questionnaire and the online link) to as many Bangladeshi people as possible who were interested in taking part in this study. As per the study plan, data collection was stopped purposively when complete responses were received from 50 participants of different age groups, between July–November 2014. Studies have been conducted in developed countries with a similarly small number of participants.^1,22–24,25^ The responders were eligible to participate in the study if they fulfilled the following criteria: aged ≥18 years; able to provide valid consent for participation; willing to participate in the study; could understand English; and had internet access. There were 20 variables in the questionnaire (written in English) to explore: what medical languages were used during medical practice in Bangladesh; if the medical language was affecting health services; and responders' opinions on what can be done to resolve the problem. When the responders clicked on the 'submit' option after completion of the questionnaire, the responses (data) were stored automatically online in a system previously built by the study investigators.

After data collection, each questionnaire was reviewed for completeness, accuracy, and internal consistency. Subsequently, the data set was converted to an SPSS file for convenient data analysis. A suitable and convenient data analysis framework was planned beforehand based on the research questions and objectives of the study. Quantitative data analyses were performed using SPSS (version 22). The reason behind choosing quantitative data analysis was that an extensive literature review noted a similar type of data analysis.^1,23–25^ Textual data analyses (by creating themes and subthemes) were performed for the open-ended questions relating to the opinions of and suggestions from the study participants. These findings were to be used to advise policymakers of what can be done to resolve the medical language barrier problem immediately, with the ultimate aim of saving patient lives.

## Results

**Figure 1.**
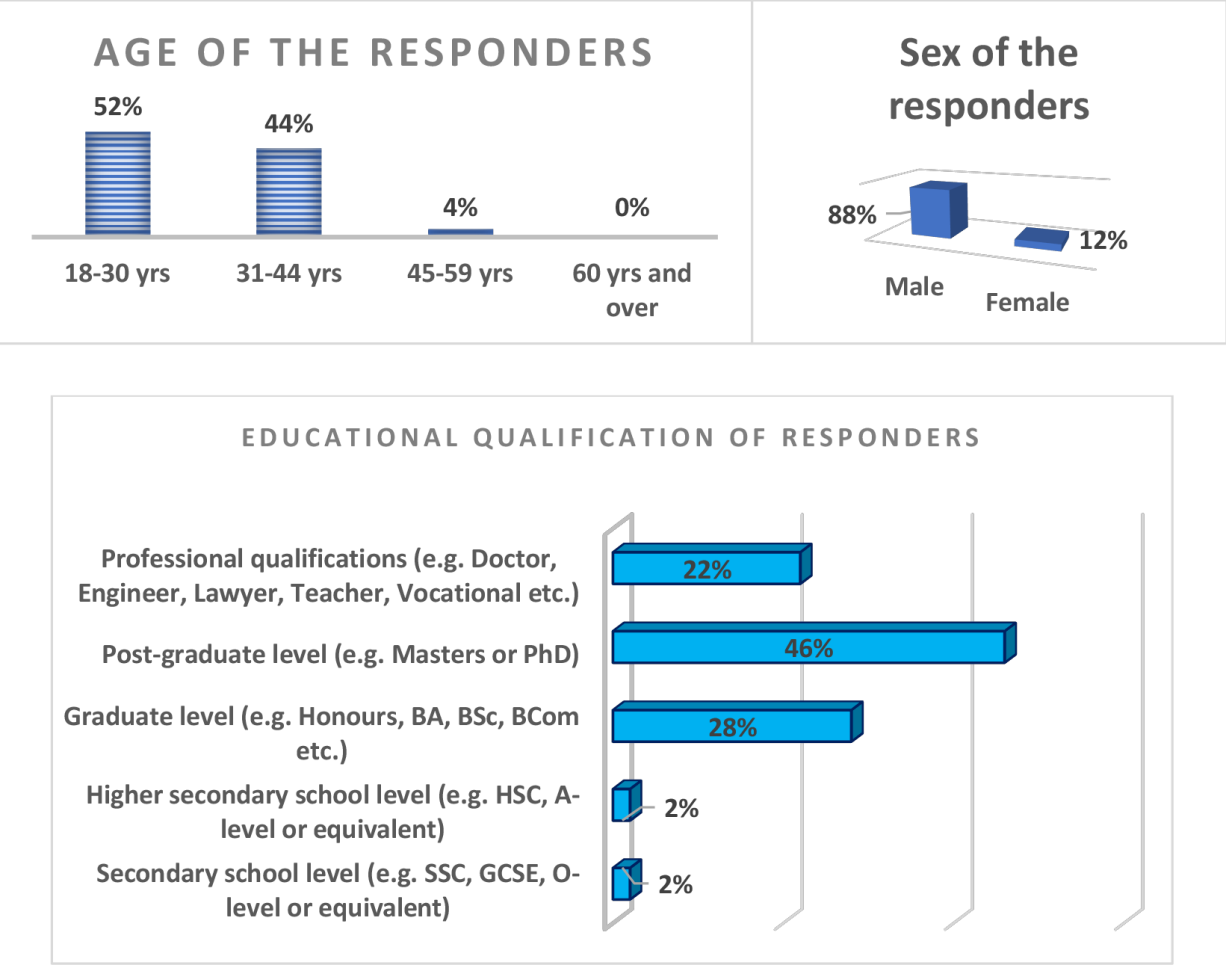
Sociodemographic characteristics of the study participants

A summary of the sociodemographic characteristics of the study participants is presented in Figure 1; more than half of the responders (52%) were in the young adult age group (18–30 years), 88% were male, and 46% of the responders were educated to post-graduate level.

**Figure 2.**
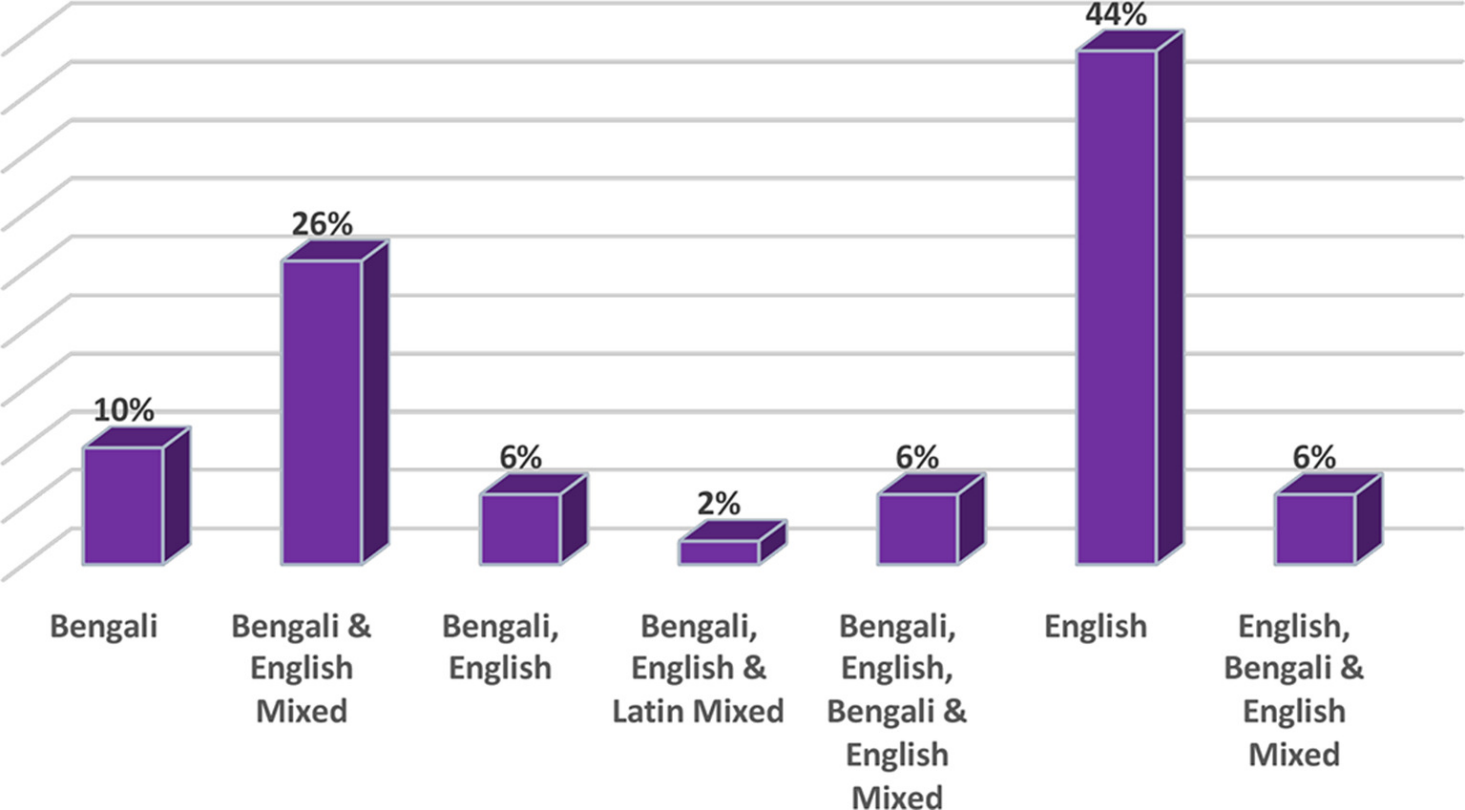
Proportion of prescriptions using each language or mix of languages, according to responders

**Figure 3.**
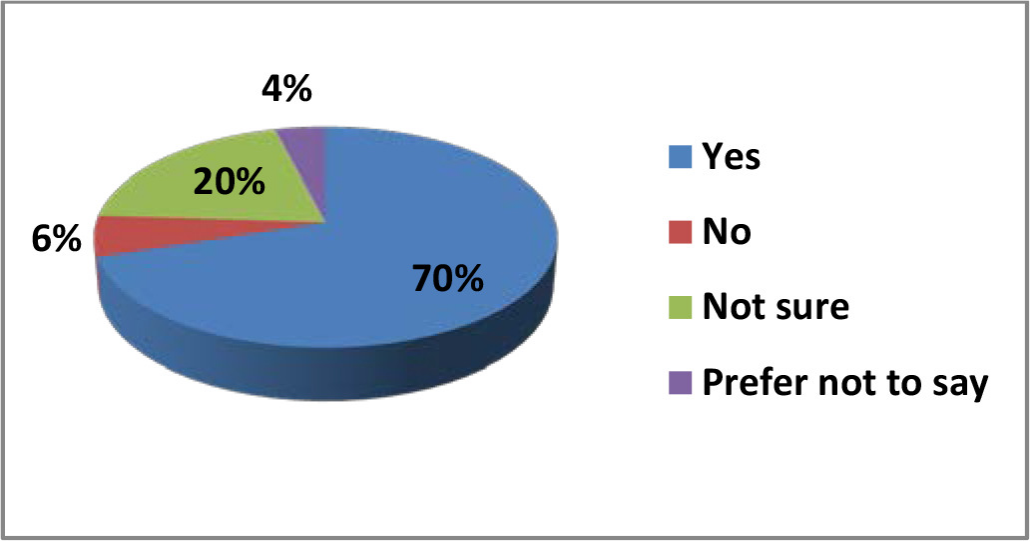
Proportion of responders that could or could not understand the language of prescriptions

Study participants were asked to give their opinion from the checklist in terms of the languages being used in prescriptions by doctors or other health service providers in Bangladesh (the options were mutually exclusive). These findings are presented in Figure 2; it can be seen that the majority of the responders (44%) identified that English was the language choice for writing prescriptions by health service providers or for discharge certificates from the hospitals, whereas 26% of responders identified that a mixture of Bengali and English was used. When asked if they could understand the language of prescriptions, 70% of the responders said that they understood the language used by the health service providers, whereas 30% could not understand the medical language (or were not sure or preferred not to say), as shown in Figure 3.

**Figure 4.**
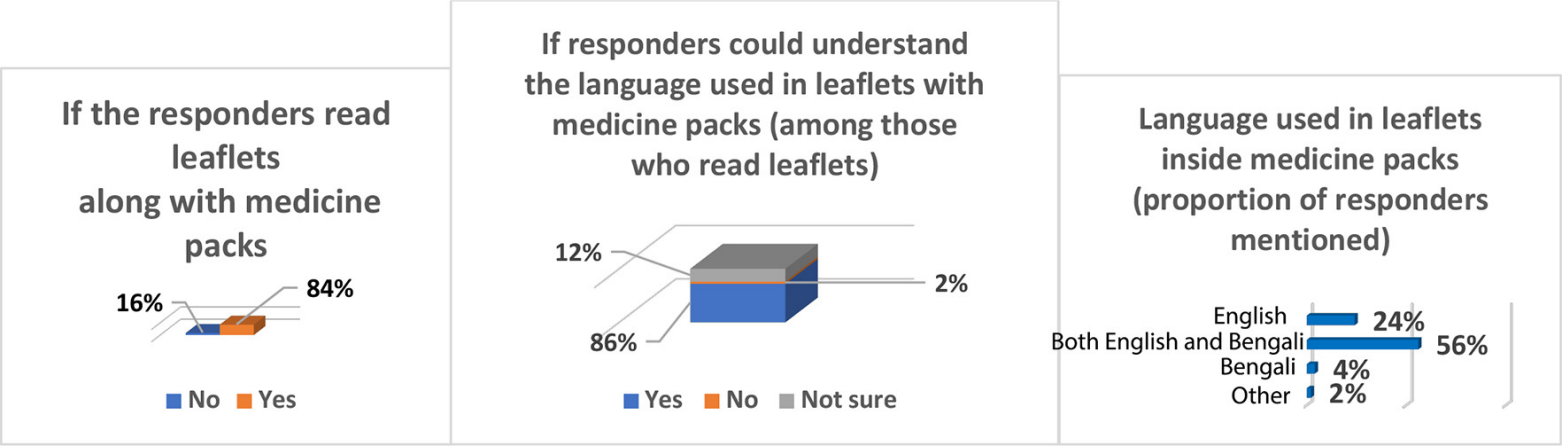
Perception of the study participants regarding the leaflets accompanying the medicine pack

**Figure 5.**
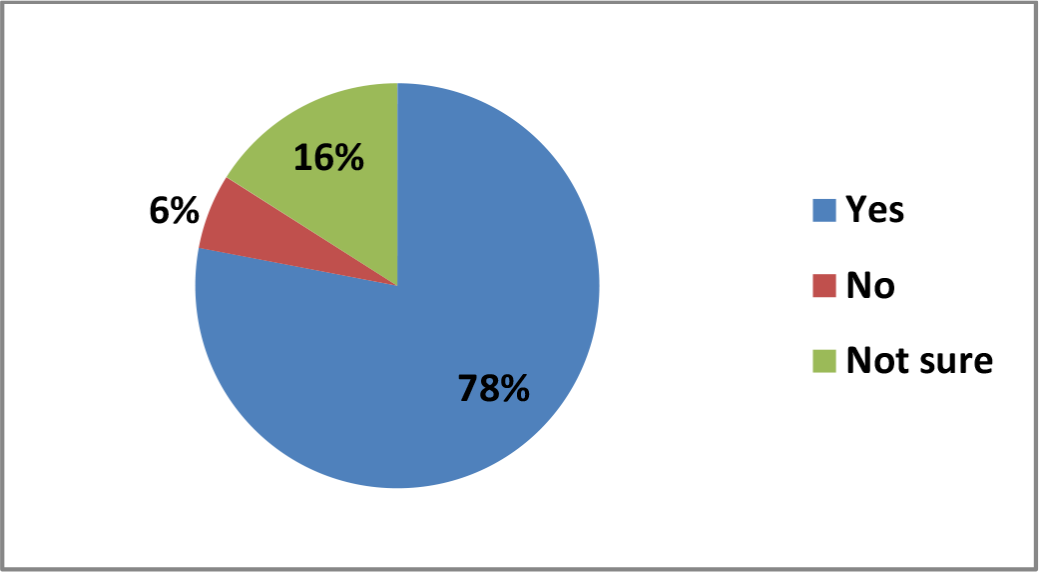
Proportion of responders that thought language affected the treatment process

When the study participants were asked whether they could understand the language used in the leaflets or instructions contained inside medicine packs, 2% said that they could not understand the language written, and 12% said they were not sure if they could understand the medical language (Figure 4). Figure 4 also illustrates the responses (in proportion) from study participants in terms of the languages used in the leaflets or instructions along with the medicine packs or inside medicine boxes, and the proportion of the responders who read the leaflets after buying medicines. More than half of the participants (56%) mentioned that both English and Bengali were the languages used to communicate the information accompanying medicine packs (for example, composition of medicines, indications, contraindications, and side effects). Of the responders, 84% said that they read the leaflets that came with the medicine packs. When they were asked if the medical language affected the treatment of patients, 78% of the responders said that medical language did affect treatment, 16% said that they were not sure, and 6% said no (Figure 5).

Table 1 shows the opinion of the responders regarding whether medical language acts as a barrier to obtaining effective health services in Bangladesh. In total, 48% of the responders said that medical language was a barrier to getting a successful outcome in providing health services in Bangladesh. When the data were analysed to see the distribution of this opinion in terms of age group, it was identified that around half of the participants of all age groups were of the opinion that medical language was a barrier in general practice.

**Table 1.** Proportion of responders mentioned that language was a barrier in health services

Age groups	Responses to the question '*Do you think that language is a barrier to get effective health services in Bangladesh?'*		
	Yes, %	No, %	Not sure, %
18–30 years	42.3	34.6	23.1
31–44 years	54.5	27.3	18.2
45 –59 years	50.0	50.0	0.0
Total	48.0	32.0	20.0

No responders were aged ≥60 years

When the responders were requested to write their opinions or suggestions with regards to what can be done to overcome the medical language barrier, a variety of opinions came out. Most of the participants suggested that health service providers used the native language of Bangladesh (Bengali), at least when writing instructions for the patients regarding taking medicines for their illnesses. A small number of responders gave the opinion that both English and Bengali should be used as a medium of providing health services:

*'Using mother tongue, that is Bengali, can easily overcome this problem.'* (Responder 5, 30 years old, Male)

' … *mother language* [Bengali] *should be used by the doctors to communicate with patients.'* (Responder 10, 42 years old, female)

*'Bengali language can be used, at least for instruction for the patients.'* (Responder 13, 28 years old, Male)

'*Language problem[s] arise with people who can't understand English. Most of them can understand Bangla* [Bengali]*, so* [the] *doctor should write the instruction in Bangla, so that patient (who can't understand English) can understand instruction.'* (Responder 45, 53 years old, Male)

Some responders advised that doctors need to give enough time to describe the illness and the medicine to the patients, so that patients are not deprived of effective health services as a result of the language barrier:

*'I think it's not possible to make the patients understand the language of doctors unless* […] *doctors make them clear about it. The doctors can make the patients clear by describing the prescription verbally.'* 

## Discussion

### Summary

Around 78% of responders in this study said that medical language was acting as an obstacle for patients when receiving treatment in Bangladesh, and about 30% of study participants could not understand the language used by the doctors or other health service providers (this includes those who were not sure or preferred not to say). Furthermore, around half of the study participants were of the opinion that misunderstanding of medical language is a barrier in obtaining effective health services in Bangladesh. These striking findings demonstrate the magnitude of the medical language barrier, which is obstructing the success of the health system in Bangladesh and raises the concern that the medical language barrier is endangering patient health.^18,20,25,26^ Therefore, it is the right time to address the burning issue of the medical language barrier in health services, which is working as a silent killer in the health sector of Bangladesh.

Most of the participants of this study suggested health service providers should use the native language when writing instructions in prescriptions; although, some responders suggested using both English and Bengali in general practice. This article also provides some policy recommendations (in the Implications for research and practice section), which have potential overcome the medical language barrier.

### Strengths and limitations

To the best of the authors' knowledge, this is the first study ever in Bangladesh that has explored barriers to access and use of healthcare services relating to medical language. The findings of the study have provided new insights, which can potentially help to improve the health system of Bangladesh and which can be scaled up in other settings around the world.

The study has several limitations that can be addressed through future research. Firstly, a small scale study was conducted owing to limited funding and time constraints, but a larger scale study could successfully focus at the crux of the problem lying inside the health system of Bangladesh. However, there are also examples of similar research around the world which was conducted with a smaller number of participants.^27–31^ Secondly, the data were collected only from educated Bangladeshi people who used the internet. As such, the views of the lower educated and illiterate people (it is predicted the problem will be higher for them), and those who do not use the internet, could not be collected or reported in this article.

### Comparison with existing literature

There is evidence from around the world that has proved that medical language is acting as a strong barrier to having a successful outcome from health services, but this issue still remained unaddressed in Bangladesh.^7,9,11,19,21,24^ A study conducted in Australia reported that communication barriers among patients and health providers can have deleterious effects on the treatment process.^25^ Considering the potentially disastrous impact of misunderstanding of medical language, this study provided evidence of medical language as a barrier to access and use of healthcare services in Bangladesh, which had never been explored in the past, as well as study participants' suggested solutions to this issue.

Usually patients can obtain information regarding medicines (for example, composition, indications, and side effects) from prescriptions (or discharge certificates from health centres), and also from the leaflets and/or instructions contained within the medicine packs.^27,32^ This study revealed that English was the language mostly used (according to 44% of the responders) in prescriptions by health service providers in Bangladesh, and leaflets accompanying medicine packs used both English and Bengali together (according to 56% of the responders). That means medical language was being delivered inconsistently to the patients and potentially causing confusion, which could endanger their health. Therefore, policymakers should take into account this huge problem to ensure the health and safety of patients.

The level of education of a country is a big factor when considering whether or not patients are able to understand medical language, and in overcoming the language barrier.^2,33^ According to UNESCO (2016), the literacy rate of Bangladesh is 72.76%.^34^ So, around 30% of people in Bangladesh cannot read and write any language (not even Bengali, the native language of Bangladesh). Furthermore, most literate people in Bangladesh are not competent in English, and can read and write in Bengali only. Therefore, this raises the question 'how can people who are uneducated read doctors' prescription, which are being written mostly in English and a mixture of other languages like Latin and Greek?' Given the scenario described by this study, it can be predicted that there are likely to be medical language barriers experienced by the rest of the population of Bangladesh, especially among those who cannot even read Bengali. For these patients, a valuable prescription is nothing more than a piece of paper if no one helps them to understand the medical language.

### Implications for research and practice

This innovative study explored the need for future research and the need for policymakers to tackle the medical language barrier in healthcare services in Bangladesh. It is believed that if the following recommendations of the studies can be followed, this underidentified problem can be resolved easily, which will help contribute to successful outcomes when receiving health care in Bangladesh:

- Health service providers should describe their instructions properly in easily understandable language to their patients during treatment so that patients do not suffer as a result of misunderstanding the medical language.
- Doctors can write the name of the medicines in English block letters and they can write instructions in the native language (Bengali), which would be understandable by all the patients who are capable of reading.
- Where applicable, sign language or illustrated information with photographs or cartoons can also be provided to convey health-related instructions or health education materials.
- Future research should explore how much verbal language is causing harm to the patients during doctor–patient interactions. Further research is also needed to explore the impact of the language barrier among illiterate people and in hard-to-reach areas.
